# Risk factors for clinical relapse in patients with ulcerative colitis who are in clinical remission but with endoscopic activity

**DOI:** 10.1002/jgh3.70011

**Published:** 2024-07-24

**Authors:** Ryosuke Horio, Jun Kato, Yuki Ohta, Takashi Taida, Keiko Saito, Miyuki Iwasaki, Yusuke Ozeki, Yushi Koshibu, Nobuaki Shu, Makoto Furuya, Yuhei Oyama, Hayato Nakazawa, Yukiyo Mamiya, Chihiro Goto, Satsuki Takahashi, Akane Kurosugi, Michiko Sonoda, Tatsuya Kaneko, Naoki Akizue, Kenichiro Okimoto, Tomoaki Matsumura, Naoya Kato

**Affiliations:** ^1^ Department of Gastroenterology Graduate School of Medicine, Chiba University Chiba Japan

**Keywords:** Mayo endoscopic score, treat to target, ulcerative colitis

## Abstract

**Background and Aim:**

The treatment strategy for patients with ulcerative colitis (UC) in clinical remission who have not achieved mucosal healing is unclear. This study aimed to determine the risk factors of relapse in patients in clinical remission with endoscopic activity.

**Methods:**

This retrospective, single‐center study included patients with UC who underwent colonoscopy (CS) and were in clinical remission with endoscopic activity. Characteristics were compared between patients who relapsed within 2 years after CS and those who did not. A Cox proportional hazards regression model was used to identify risk factors contributing to clinical relapse. Recent worsening in bowel symptoms was defined as increase in bowel frequency and/or increase in abdominal pain within approximately 1 month based on the descriptions in the medical charts.

**Results:**

This study regarded 142 patients in clinical remission with an endoscopic activity of Mayo endoscopic subscore (MES) of ≥1 as eligible, and 33 (23%) patients relapsed during the observation period. Recent worsening of bowel symptoms was a significant risk factor for clinical relapse (hazard ratio [HR]: 3.02, 95% confidence interval [CI]: 1.34–6.84). This was particularly evident in patients with MES of 2 (HR: 5.16, 95% CI: 1.48–18.04), whereas no risk factors were identified in patients with MES of 1. The presence or absence of therapeutic intervention just after CS did not significantly affect clinical relapse.

**Conclusion:**

Recent worsening in bowel symptoms was a significant risk factor for clinical relapse in patients with UC who were in clinical remission with endoscopic activity.

## Graphical abstract

This study revealed that recent worsening in bowel symptoms was a significant risk factor for clinical relapse in ulcerative colitis [UC] patients in clinical remission but without mucosal healing. This trend is particularly evident in patients with higher endoscopic activity (Mayo endoscopic subscore [MES] of 2) but not in those with lower endoscopic activity (MES of 1). Therefore, patients in clinical remission should be thoroughly interviewed for symptoms and therapeutic intervention should be cautiously considered for those with endoscopic activity.

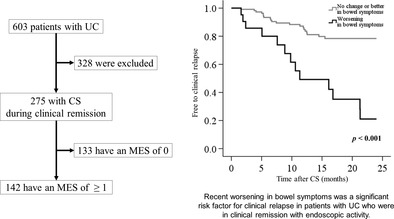

## Introduction

Ulcerative colitis (UC) is a chronic inflammatory disease of unknown etiology that causes the colonic mucosal inflammation to become serially distended from the rectal side, frequently forming erosions and ulcers.^1^ In recent years, UC prevalence in Asian countries, including Japan, has been rapidly increasing.^2^ Symptoms, such as abdominal pain, diarrhea, and bloody stools, have significantly affected the quality of life of patients and made their social life difficult.^3^

Traditionally, the treatment strategy for inflammatory bowel diseases, including UC, has aimed to improve clinical symptoms, and induce and maintain clinical remission. However, several reports have recently indicated that achieving mucosal healing reduces the risk of clinical relapse^4^, ^5^, ^6^, ^7^ and proposed mucosal healing as a long‐term therapeutic target.^8^ Conversely, the treatment strategy for patients with UC who are in clinical remission but with endoscopic activity appeared difficult, because some of these patients experience relapse but others do not. Concerns arose regarding the risk of adverse effects and increased costs associated with therapeutic intervention for such patients. The optimal treatment strategy for these patients should be targeted therapeutic intervention for only those who are likely to relapse. Hence, exploring risk factors for clinical relapse in patients with UC who are in clinical remission but with endoscopic activity is a crucial clinical issue.

Therefore, this study investigated risk factors for clinical relapse in patients with UC who were in clinical remission but had not achieved mucosal healing. Our results would help determine patients for whom treatment should be intensified and cause better treat‐to‐target strategy for UC.

## Methods

### Study design and patients

This retrospective observational study of patients with UC in clinical remission with endoscopic activity investigated relapse and risk factors for relapse in such patients. This study included all patients with UC who had regular visits to Chiba University Hospital and underwent colonoscopy (CS) at least 3 months after achieving clinical remission from April 2011 to September 2022. UC was diagnosed according to the European Crohn's and Colitis Organisation consensus guidelines.^9^ Clinical remission was defined as a partial Mayo (pMayo) score of ≤2 with a rectal bleeding score of zero,^10^ and pMayo was determined retrospectively by one of the authors according to the descriptions of the medical charts. This study excluded patients who were not in clinical remission or were receiving corticosteroids or calcineurin inhibitors upon CS, and those who had a change of treatment within 3 months before CS, as well as those who had failed insertion into the cecum at CS.

Colonoscopic results were evaluated using Mayo endoscopic subscore (MES),^10^ and locations of the observed MES were noted. Two investigators of this study (Y.O. and T.T.) reviewed the endoscopic images to confirm the MES, in addition to the MES evaluated during CS. MES of 0 indicated mucosal healing. This study aimed to investigate the prognostic outcomes of patients in clinical remission with endoscopic activity; thus, those who had colonoscopic results of MES of 0 were excluded.

Patients who met the inclusion criteria for this study were followed up to the time of relapse for those with relapse, or to the time of the outpatient visit immediately before 2 years after CS or until January 2023 for those with no relapse. All patients had regular outpatient visits every 1–3 months after CS. Relapse was a flare of clinical symptoms of UC that is pMayo ≥3 and requiring any medical treatment. The first episode alone was included if patients had two or more episodes of meeting the inclusion criteria.

### Outcomes and data collections

The primary outcome was to elucidate the clinical relapse rate and identify risk factors for clinical relapse in patients with UC who were in clinical remission but had not achieved mucosal healing.

All data were collected from medical charts, as well as endoscopic images and reports. The following data at CS were obtained: age, sex, disease duration, extent of disease, medications, relapse incidences up to the time of CS, recent changes in bowel symptoms, therapeutic intervention administration just after CS, and laboratory values, including white blood cell count (WBC) (/μL), hemoglobin (Hb) (g/dL), platelet count (Plt) (10^4^/μL), serum albumin (Alb) (g/dL), and C‐reactive protein (CRP) (mg/dL). Recent worsening in bowel symptoms was defined as increase in bowel frequency and/or increase in abdominal pain within approximately one month based on the descriptions in the medical charts. This information was determined by one of the authors who did not know the outcome of whether or not a relapse had occurred.

### Statistical analysis

The Mann–Whitney *U*‐test and Fisher's exact test or Pearson's chi‐square test for continuous and categorical variables, respectively, were used to compare patient groups. Kaplan–Meier survival curves were used to calculate the overall survival to clinical relapse, and the log‐rank test was applied to compare survival rates between groups. Additionally, significant factors contributing to clinical relapse were determined using a Cox proportional hazards regression model. Evaluated variables include sex, age, disease duration, extent of disease, relapse incidences, serum laboratory values (Alb, WBC, Hb, Plt, and CRP), recent changes in bowel symptoms, therapeutic intervention administration just after CS, MES, and endoscopic activity locations. Factors with a *P*‐value of <0.05 in the univariate analysis were incorporated into multivariate analysis as explanatory factors. A *P*‐value of <0.05 was considered significant. Statistical Package for the Social Sciences version 29.0 (IBM, Chicago, IL, USA) was used for all statistical data analyses.

### Ethical considerations

The Institutional Ethics Committee under the principles of the Declaration of Helsinki and the current code of ethics (No. 2898) approved this study. Written informed consent from the patient was waived because the data were anonymously and retrospectively analyzed.

## Results

### Patient characteristics

**Figure 1:**
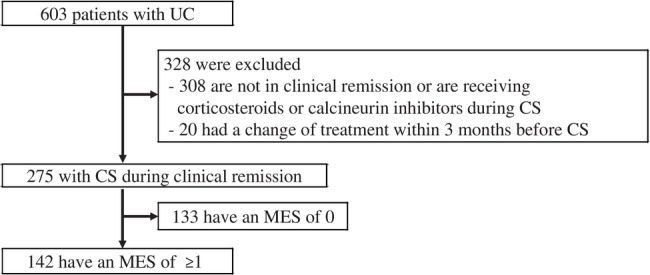
Flowchart of the study population. The analysis included 142 patients in clinical remission with endoscopic activity of Mayo endoscopic subscore (MES) of ≥1. CS, colonoscopy; UC, ulcerative colitis.

A total of 603 patients with UC who had regular visits to Chiba University Hospital underwent CS from April 2011 to September 2022. Of the 603 patients, 308, who were not in clinical remission or were receiving corticosteroids or calcineurin inhibitors during CS, and 20, who had a change of treatment within 3 months before CS, were excluded, as well as 133 patients with MES of 0. Ultimately, the analysis included 142 patients in clinical remission with endoscopic activity of MES of ≥1 (Fig. 1).

**Figure 2:**
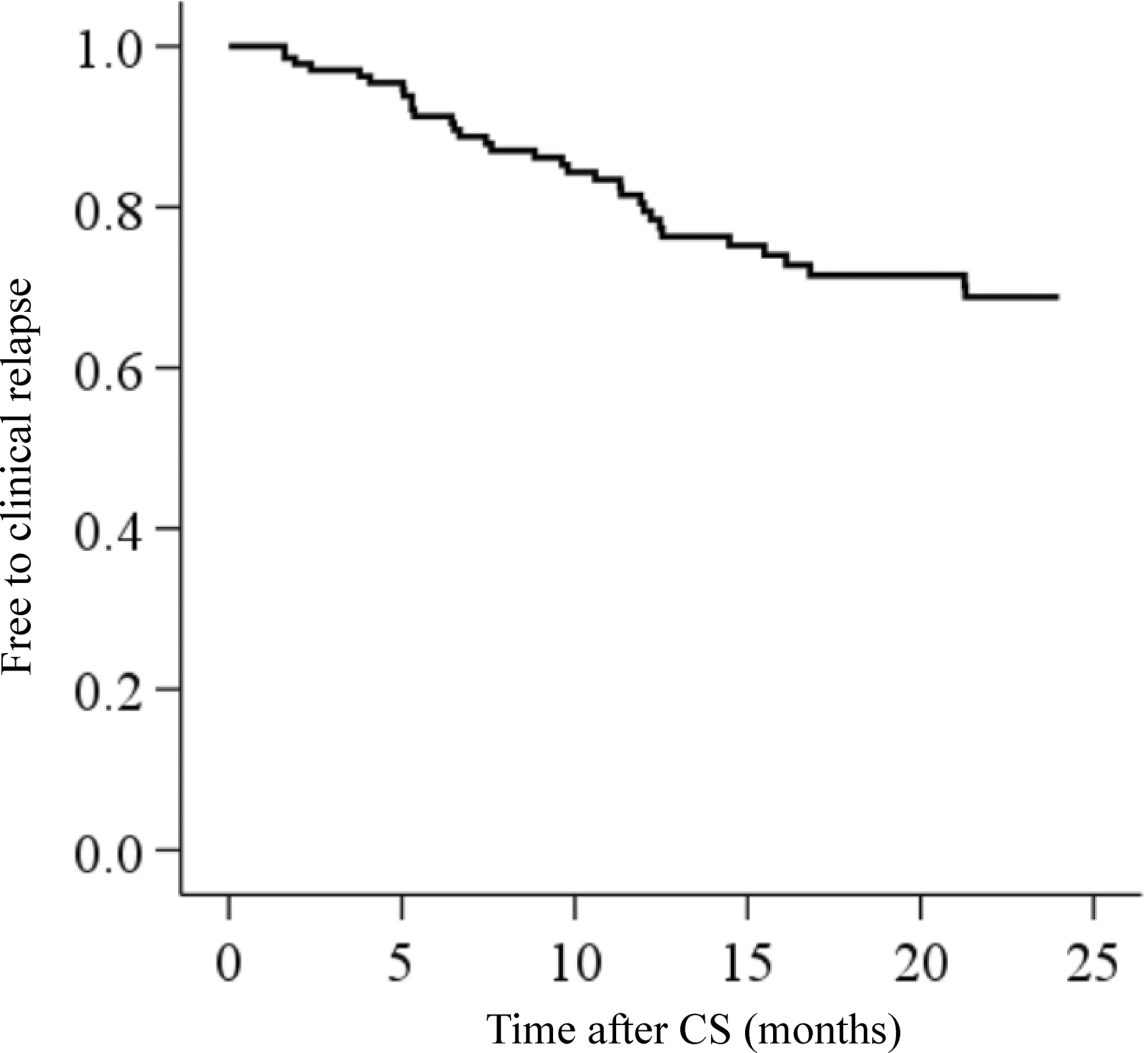
Kaplan–Meier curves for clinical relapse in 142 patients. Of the 142 patients, 33 (23%) relapsed. CS, colonoscopy.

Table 1 shows the baseline characteristics of the 142 patients; 88 (62%) patients were male, the median age was 38 years (interquartile range [IQR]: 26–48 years), and the disease duration was 4.0 years (IQR: 1.8–7.2 years). Immunomodulators and biologics (Bio)/Janus kinase (JAK) inhibitors were used in 44 (31%) and 34 (24%) cases, respectively. Therapeutic intervention just after CS was performed in 41 (29%) patients; 23 (56%) with 5‐aminosalicylates (5‐ASA), 12 (29%) with corticosteroids, 3 (7%) with immunomodulators, 1 (2%) with Bio/JAK inhibitors, and 2 (5%) with others. Regarding the recent changes in bowel symptoms, 121 (85%) patients reported no or better changes, while 21 (15%) reported worsening. All patients with recent changes in bowel symptoms had increased bowel frequency and five (24%) of them complained of increased abdominal pain. Of those with increased abdominal pain, three patients relapsed and two patients did not (*P* = 0.330). The endoscopic activity was MES of 1 and 2 in 96 (68%) and 46 (32%) patients, respectively. Of these patients, 33 (23%) relapsed during the observation period (Fig. 2). Medications of therapeutic intervention at clinical relapse were 5‐ASA in 6 patients (18%), corticosteroids in 19 patients (58%), Bio/JAK inhibitors in 6 patients (18%), and others in 2 patients (6%).

**Table 1:** Baseline characteristics of 142 patients in this study

	*n* = 142
Sex, male:female (male %)	88:54 (62)
Age, years (IQR)	38 (26–48)
Duration of disease, years (IQR)	4.0 (1.8–7.2)
Extent of disease (%)	
Extensive	84 (59)
Left‐sided	38 (27)
Rectal	12 (8)
Right‐sided	8 (6)
Number of relapses (past 2 years)	
0 (%)	44 (31)
1 (%)	67 (47)
≥2 (%)	31 (22)
Alb, g/dL (IQR)	4.4 (4.2–4.6)
WBC, /μL (IQR)	6100 (4900–7400)
Hb, g/dL (IQR)	13.9 (12.9–15.3)
Plt, 10^4^/μL (IQR)	26.6 (22.0–29.9)
CRP, mg/dL (IQR)	0.06 (0.00–0.13)
Medication (%)	
5‐ASA	
Oral treatment	120 (85)
Topical treatment	38 (27)
Immunomodulators	44 (31)
Bio/JAK inhibitors	34 (24)
Others	6 (4)
Therapeutic intervention just after CS (%)	41 (29)
Recent changes in bowel symptoms	
No change or better	121 (85)
Worsening	21 (15)
MES (%)	
1	96 (68)
2	46 (32)
Observed MES locations (%)	
Sigmoid colon–rectum	79 (56)
Cecum–descending colon	29 (20)
Both	34 (24)
Observation period (months, IQR)	13.0 (6.5–24.0)

5‐ASA, 5‐aminosalicylates; Alb, albumin; Bio, biologics; CRP, C‐reactive protein; CS, colonoscopy; Hb, hemoglobin; IQR, interquartile range; JAK, Janus kinase; MES, Mayo endoscopic subscore; Plt, platelet; WBC, white blood cell.

Table 2 shows the comparison of the characteristics between patients with and without relapse. Patients with relapse were significantly younger than those without relapse (32 [22–42] *vs* 39 [30–51], *P* = 0.019). Sex, disease duration, extent of disease, relapse incidences, laboratory values, medications, and observed MES locations indicated no significant differences between the two groups. Recent worsening of symptoms was more frequently observed in patients with than in those without relapse (39% *vs* 7%, *P* < 0.001). Therapeutic intervention just after CS was significantly more frequently provided in patients with relapse (58% *vs* 20%, *P* < 0.001). Endoscopic activity was significantly higher in patients with relapse (MES of 1: 42% *vs* 75%; MES of 2: 58% *vs* 25%; *P* < 0.001).

**Table 2:** Comparison of characteristics between patients with and without relapse

	Patients with relapse (*n* = 33)	Patients without relapse (*n* = 109)	*P*
Sex, male:female (male %)	21:12 (64)	67:42 (61)	0.822
Age, years (IQR)	32 (22–42)	39 (30–51)	0.019
Disease duration	4.0 (1.9–5.8)	4.0 (1.9–8.4)	0.615
Extent of disease (%)			0.104
Extensive	22 (67)	62 (57)	
Left‐sided	6 (18)	32 (29)	
Rectal	1 (3)	11 (10)	
Right‐sided	4 (12)	4 (4)	
Number of relapses (past 2 years)			0.354
0 (%)	7 (21)	37 (34)	
1 (%)	17 (52)	50 (46)	
≥2 (%)	9 (27)	22 (20)	
Alb, g/dL (IQR)	4.5 (4.3–4.6)	4.3 (4.1–4.6)	0.050
WBC, /μL (IQR)	5900 (4800–7700)	6200 (4900–7350)	0.982
Hb, g/dL (IQR)	14.2 (12.5–15.9)	13.9 (13.1–15.2)	0.536
Plt, 10^4^/μL (IQR)	25.5 (23.7–29.7)	26.7 (21.3–29.9)	0.622
CRP, mg/dL (IQR)	0.03 (0.00–0.20)	0.06 (0.05–0.12)	0.963
Medication (%)			
5‐ASA			
Oral treatment	29 (88)	91 (83)	0.541
Topical treatment	8 (24)	30 (28)	0.709
Immunomodulators	10 (30)	34 (31)	0.923
Bio/JAK inhibitors	7 (21)	27 (25)	0.675
Others	2 (6)	4 (4)	0.550
Therapeutic intervention just after CS (%)	19 (58)	22 (20)	<0.001
Recent changes in bowel symptoms			<0.001
No change or better	20 (61)	101 (92)	
Worsening	13 (39)	8 (7)	
MES (%)			<0.001
1	14 (42)	82 (75)	
2	19 (58)	27 (25)	
Observed MES Locations (%)			0.338
Sigmoid colon–rectum	19 (58)	60 (55)	
Cecum–descending colon	4 (12)	25 (23)	
Both	10 (30)	24 (22)	

5‐ASA, 5‐aminosalicylates; Alb, albumin; Bio, biologics; CRP, C‐reactive protein; CS, colonoscopy; Hb, hemoglobin; IQR, interquartile range; JAK, Janus kinase; MES, Mayo endoscopic subscore; Plt, platelet; WBC, white blood cell.

### Prognostic factors for clinical relapse

A Cox proportional hazards model was used to analyze factors contributing to clinical relapse (Tables 3, 4, 5). Multivariate analysis revealed that recent worsening in bowel symptoms was a significant risk factor for clinical relapse (hazard ratio [HR]: 3.02, 95% confidence interval [CI]: 1.34–6.84) (Table 3). The analysis limited to the patients with colonoscopic findings of MES of 1 revealed no significant factors in the multivariate analysis (Table 4). In contrast, the analysis limited to the patients with colonoscopic findings of MES of 2 revealed that recent worsening in bowel symptoms was a significant risk factor for clinical relapse (HR: 5.16, 95% CI: 1.48–18.04) (Table 5). The presence or absence of therapeutic intervention just after CS did not significantly affect clinical relapse.

**Table 3:** Analysis of factors predicting clinical relapse in 142 patients with a Mayo endoscopic subscore of ≥1

	Univariate			Multivariate		
	HR	95% CI	*P*	HR	95% CI	*P*
Sex (male)	1.14	0.56–2.31	0.724			
Age (<38 years old)	1.84	0.91–3.75	0.091			
Disease duration (≥4 years)	1.02	0.52–2.03	0.945			
Extent of disease (extensive)	1.33	0.64–2.73	0.446			
Relapse in past 2 years	1.84	0.80–4.25	0.151			
Alb (<4.5 g/dL)	0.52	0.26–1.03	0.061			
WBC (≥6000/μL)	0.98	0.50–1.95	0.964			
Hb (<14.0 g/dL)	0.73	0.37–1.44	0.363			
Plt (≥25.0 × 10^4^/μL)	1.16	0.58–2.29	0.678			
CRP (≥0.05 mg/dL)	0.91	0.46–1.81	0.797			
Therapeutic intervention just after CS	3.60	1.80–7.17	<0.001	1.90	0.82–4.39	0.136
Recent worsening in bowel symptoms	4.98	2.47–10.03	<0.001	3.02	1.34–6.84	0.008
MES 2	2.96	1.48–5.90	0.002	1.98	0.94–4.20	0.074
Observed MES locations (cecum–descending colon)	0.93	0.46–1.85	0.827			

Alb, albumin; CI, confidence interval; CRP, C‐reactive protein; CS, colonoscopy; Hb, hemoglobin; HR, hazard ratio; MES, Mayo endoscopic subscore; Plt, platelet; WBC, white blood cell.

**Table 4:** Analysis of factors predicting clinical relapse in 96 patients with a Mayo endoscopic subscore of 1

	Univariate			Multivariate		
	HR	95% CI	*P*	HR	95% CI	*P*
Sex (male)	1.05	0.35–3.15	0.925			
Age (<38 years old)	1.42	0.49–4.10	0.516			
Disease duration (≥4 years)	0.82	0.28–2.36	0.713			
Extent of disease (Extensive)	1.22	0.41–3.64	0.724			
Relapse in past 2 years	1.70	0.47–6.09	0.417			
Alb (<4.5 g/dL)	0.31	0.10–0.93	0.037			
WBC (≥6000/μL)	0.77	0.27–2.22	0.626			
Hb (<14.0 g/dL)	0.91	0.32–2.61	0.866			
Plt (≥25.0 × 10^4^/μL)	0.63	0.21–1.87	0.403			
CRP (≥0.05 mg/dL)	0.47	0.15–1.49	0.198			
Therapeutic intervention just after CS	2.27	0.76–6.79	0.141	2.14	0.70–6.57	0.184
Recent worsening in bowel symptoms	1.86	0.41–8.31	0.419	1.49	0.32–6.96	0.609
Observed MES locations (cecum–descending colon)	1.32	0.46–3.77	0.600			

Alb, albumin; CI, confidence interval; CRP, C‐reactive protein; CS, colonoscopy; Hb, hemoglobin; HR, hazard ratio; MES, Mayo endoscopic subscore; Plt, platelet; WBC, white blood cell.

**Table 5:** Analysis of factors predicting clinical relapse in 46 patients with a Mayo endoscopic subscore of 2

	Univariate			Multivariate		
	HR	95% CI	*P*	HR	95% CI	*P*
Sex (male)	1.49	0.59–3.80	0.396			
Age (<38 years old)	2.25	0.85–5.94	0.102			
Disease duration (≥4 years)	1.20	0.49–2.96	0.692			
Extent of disease (Extensive)	1.41	0.53–3.73	0.492			
Relapse in past 2 years	2.17	0.72–6.59	0.170			
Alb (<4.5 g/dL)	0.74	0.30–1.84	0.512			
WBC (≥6000/μL)	1.25	0.51–3.09	0.627			
Hb (<14.0 g/dL)	0.53	0.21–1.33	0.179			
Plt (≥25.0 × 10^4^/μL)	1.47	0.56–3.87	0.437			
CRP (≥0.05 mg/dL)	1.13	0.45–2.88	0.796			
Therapeutic intervention just after CS	3.42	1.23–9.56	0.019	1.32	0.33–5.31	0.698
Recent worsening in bowel symptoms	6.14	2.42–15.58	<0.001	5.16	1.48–18.04	0.010
Observed MES locations (cecum–descending colon)	0.67	0.26–1.70	0.395			

Alb, albumin; CI, confidence interval; CRP, C‐reactive protein; Hb, hemoglobin; HR, hazard ratio; MES, Mayo endoscopic subscore; Plt, platelet; WBC, white blood cell.

### Prognostic impact of recent worsening in bowel symptoms on patients with UC in clinical remission

**Figure 3:**
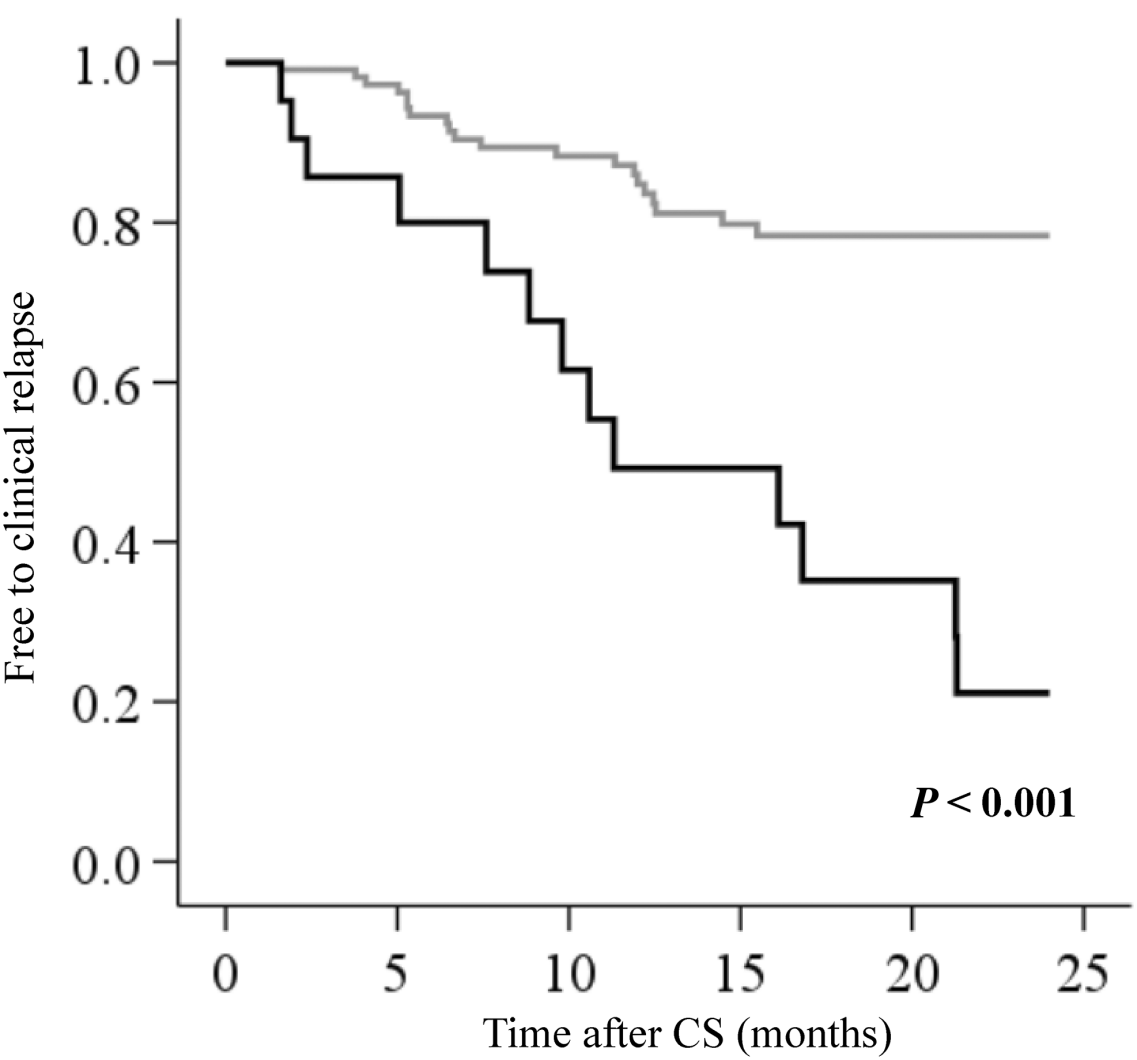
Kaplan–Meier curves for clinical relapse in 142 patients with a Mayo endoscopic score of ≥1. Patients with recent worsening in bowel symptoms were significantly more likely to have clinical relapse (*P* < 0.001). (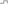), No change or better in bowel symptoms; (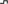), worsening in bowel symptoms. CS, colonoscopy.

**Figure 4:**
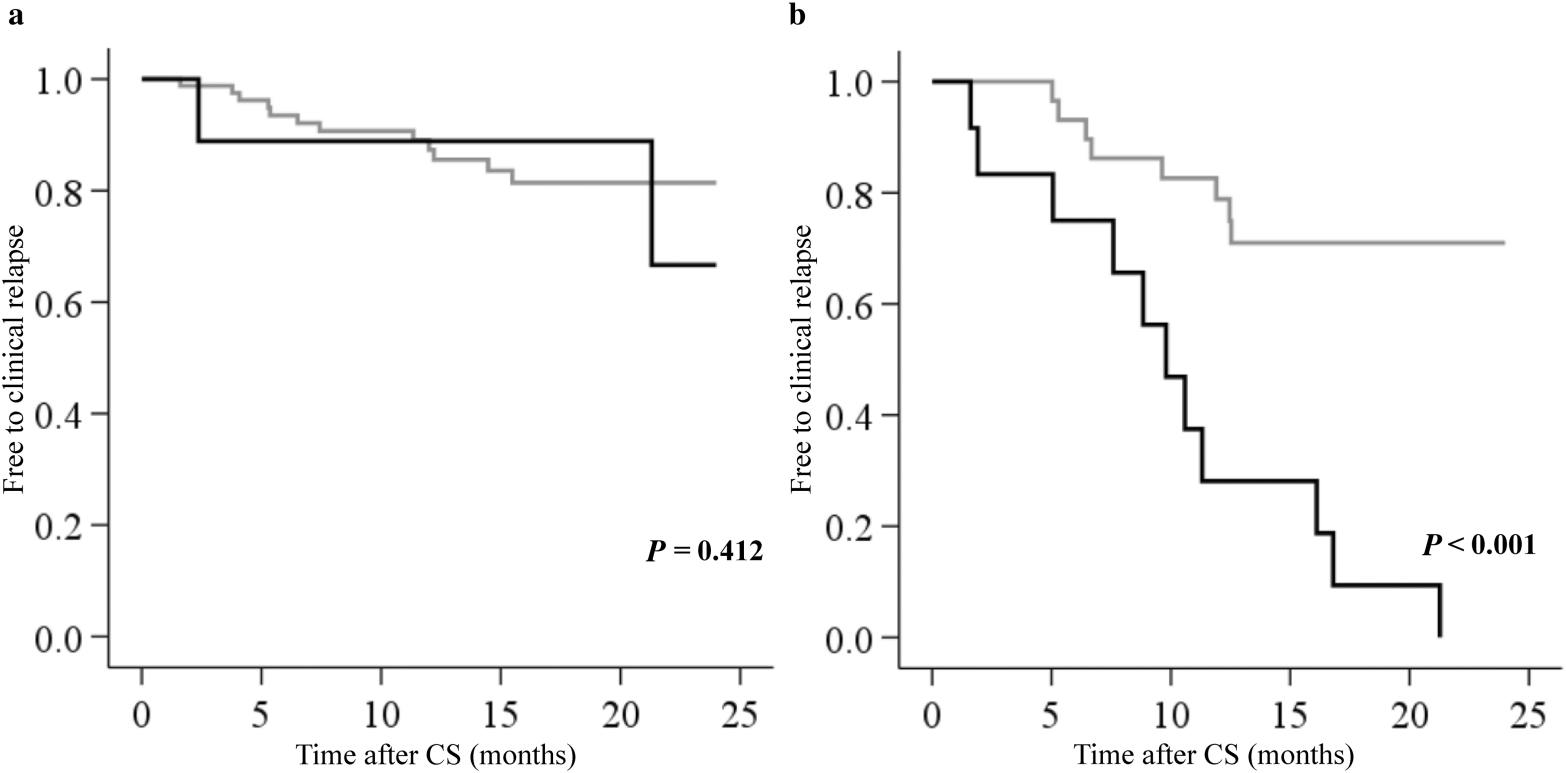
Kaplan–Meier curves for clinical relapse in 96 patients with a Mayo endoscopic score (MES) of 1 (a) and 46 patients with an MES of 2 (b). No significant difference was observed in clinical relapse between patients with and without recent worsening of symptoms in patients with MES of 1 (*P* = 0.412), while in patients with MES of 2, those with recent worsening in bowel symptoms were more likely to have clinical relapse (*P* < 0.001). (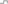), No change or better in bowel symptoms; (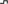), worsening in bowel symptoms. CS, colonoscopy.

The Kaplan–Meier curves showed the occurrence of clinical relapse according to the presence or absence of recent worsening in bowel symptoms (Fig. 3). Patients with recent worsening bowel symptoms were more likely to have clinical relapse (*P* < 0.001, log‐rank test). Subanalysis according to MES revealed no significant difference in clinical relapse between the two groups in patients with MES of 1 (*P* = 0.412) (Fig. 4a), while patients with MES of 2 with recent worsening bowel symptoms were more likely to have clinical relapse (*P* < 0.001) (Fig. 4b).

## Discussion

This study revealed that recent worsening in bowel symptoms was a significant risk factor for clinical relapse in patients with UC who were in clinical remission but had not achieved mucosal healing. This trend is particularly evident in patients with higher endoscopic activity (MES of 2) but not in patients with lower endoscopic activity (MES of 1). These results indicate that even if UC patients are in clinical remission by definition, if they are endoscopically active, patients with recently worsening bowel symptoms are more likely to relapse, especially in those with high endoscopic activity.

The long‐term treatment goal for UC has shifted from traditional clinical remission to mucosal healing in the treat‐to‐target strategies proposed by the International Organization for the Study of Inflammatory Bowel Diseases.^8^ This is because many studies have revealed that mucosal healing reduces the risk of clinical relapse,^4^, ^5^, ^6^, ^7^ and meta‐analyses have demonstrated that achieving mucosal healing reduces the risk of clinical relapse.^11^ A randomized controlled trial of Crohn's disease revealed that normalization of biomarkers and endoscopic findings, in addition to clinical symptoms, reduces the risk of recurrence.^12^, ^13^ However, the validity of treatment intensification in UC to pursue mucosal healing in patients with clinical remission has not fully been verified. Some patients with UC who are in clinical remission without mucosal healing do not relapse, and concerns arise about the risk of side effects and increased costs associated with therapeutic interventions for such patients. Therefore, investigating the factors that contribute to clinical relapse is beneficial.

This study identified recent worsening in bowel symptoms as a risk factor for clinical relapse. Based on these results, even for patients in clinical remission, we have to be more sensitive to the change in their symptoms at the interview during their visit. Our findings are clinically very relevant in terms of identifying a group that truly needs therapeutic intervention in patients in clinical remission with endoscopic activity. Hence, we propose that the definition of clinical remission should include the trend of change of symptoms, in addition to the absolute value of the pMayo score.

Yokoyama et al. revealed a higher relapse rate in patients with UC in clinical remission with an MES of 2 compared with those with an MES of 1,^6^ and this was congruent with our results. However, the necessity of treatment intervention for all patients with MES of 2 is not necessarily warranted, as individuals with an MES of 2 do not always experience relapse. Thus, Asonuma et al. reported that the residual distal short‐segment inflammation, even MES of 2 or 3, was not a risk factor for major relapse in patients with UC who had extensive colitis in clinical remission.^14^ This study showed that the patients with no or better changes in recent bowel symptoms had significantly fewer relapses than patients with recent worsening in bowel symptoms even in patients with MES of 2. Therefore, our results indicate that most patients in clinical remission with MES of 1 do not require therapeutic intervention, and therapeutic intervention even in patients in clinical remission with MES of 2 should be carefully considered based on the recent trend of bowel symptoms.

Our study revealed that therapeutic intervention just after CS in patients with UC who were in clinical remission but had not achieved mucosal healing was not identified as the factor that affected clinical relapse. In contrast, Fukuda et al. revealed that therapeutic intervention for patients in clinical remission with endoscopic activity MES of 1 may be effective for clinical relapse prevention.^15^ However, the 1‐year relapse rate of the patients without therapeutic intervention in their study was 52%, which was much higher than ours (11%). This discrepancy could be associated with the difference in patient characteristics, for example, the much higher rate of patients with Bio/JAK inhibitors in our study (24% *vs* 7%). Therefore, our patients were not likely to relapse, and the means of intervention may be limited. In addition, 23 (56%) of the treatment interventions were 5‐ASA, which might be weak for changing the disease course of these patients. Therapeutic intervention for patients in clinical remission but with endoscopic activity should be considered more cautiously because the use of Bio/JAK inhibitors has become more prevalent in recent years.

Laboratory values, including CRP, Plt, and Alb, have predicted endoscopic activity,^16^ while this study identified them as not predictors of clinical relapse in patients with UC who were in clinical remission but had not achieved mucosal healing. However, fecal calprotectin (FC) and leucine‐rich alpha‐2 glycoprotein (LRG), which were not measured in this study, have correlated better with endoscopic activity and were more likely to predict clinical relapse than CRP.^17^, ^18^ Therefore, the strategy of providing therapeutic intervention for patients with recent worsening of bowel symptoms without observation via CS may deserve consideration in cases with high FC or LRG, because most patients in clinical remission do not wish to receive CS due to high physical and economic burdens.

This study has an inevitable limitation accompanied by its retrospective and single‐center study. pMayo was determined retrospectively by one of the authors according to the descriptions of the medical charts, and therefore could sometimes be not quite accurate, particularly in the item of the physician's global assessment. Similarly, recent worsening in bowel symptoms might have been missed if descriptions in the medical charts were not accurate. Only insufficient information was available on the impact of other factors, such as bowel symptoms other than increased stool frequency and abdominal pain, medication adherence, and lifestyle modification, on the risk of clinical relapse. In addition, the possibility of unintentional selection bias in patient selection cannot be completely ruled out. However, our results regarding the risk of relapse in patients in clinical remission with endoscopic activity are unique and could be generalized for patients with UC seen at any facility in any country. Moreover, we are planning a prospective observational study to validate our findings regarding recent worsening of symptoms and to explore additional risk factors.

In conclusion, recent worsening in bowel symptoms was a significant risk factor for clinical relapse in patients with UC who were in clinical remission but had not achieved mucosal healing. Our results confirmed that even patients in clinical remission by definition should be thoroughly interviewed for symptoms and that therapeutic intervention should be cautiously considered for patients in clinical remission with endoscopic activity.

## Patient consent statement

Written informed consent from the patient was waived because the data were anonymously and retrospectively analyzed.

## Data Availability

The data underpinning the conclusions of this study are accessible upon request from the corresponding author.
